# Zinc Oxide Nanoparticles as Next-Generation Feed Additives: Bridging Antimicrobial Efficacy, Growth Promotion, and Sustainable Strategies in Animal Nutrition

**DOI:** 10.3390/nano15131030

**Published:** 2025-07-02

**Authors:** Jiayi Yang, Dongwei Xiong, Miao Long

**Affiliations:** Key Laboratory of Livestock Infectious Diseases, Ministry of Education, and Key Laboratory of Ruminant Infectious Disease Prevention and Control (East), Ministry of Agriculture and Rural Affairs, College of Animal Science and Veterinary Medicine, Shenyang Agricultural University, 120 Dongling Road, Shenyang 110866, China; 2023220648@stu.syau.edu.cn (J.Y.); 2022200183@stu.syau.edu.cn (D.X.)

**Keywords:** zinc oxide, nanoparticles, biological functions, animal production, feed additives

## Abstract

Zinc oxide nanoparticles (ZnO NPs) have attracted significant attention due to their wide-ranging applications in animal production, largely because of their notable biocompatibility, low toxicity, and strong antimicrobial activity. These properties make ZnO NPs a promising substitute for traditional antibiotics. Their application could address the growing concern of antibiotic resistance in livestock industries. This review examines the unique physicochemical characteristics of ZnO NPs, including their nanoscale size and high surface area, which contribute to their biological functionality. Emphasis is placed on green synthesis methods that minimize environmental impact while producing high-quality ZnO NPs. In animal farming, ZnO NPs play a crucial role not only in promoting growth and improving immune responses, but also in enhancing meat and egg quality. Additionally, this review discusses the environmental and safety implications of ZnO NPs, considering their sustainable application potential in future animal production practices, aimed at fostering a more eco-friendly and efficient livestock sector.

## 1. Introduction

As the antibiotic resistance crisis intensifies, the global livestock industry faces unprecedented challenges. The overuse of antibiotics has not only led to the spread of resistant bacteria but also poses significant threats to animal health and human food safety. Therefore, finding alternatives to antibiotics has become an urgent issue in animal production. The rapid development of nanotechnology has provided new solutions to this problem, with nano-sized zinc oxide (ZnO NPs) showing unique potential in the field of animal nutrition.

Due to its excellent biocompatibility, high surface activity, and multifunctionality, ZnO NPs have become a hot topic in recent research. The small size of ZnO NPs (ranging from 1 to 100 nm) gives them a large specific surface area, which allows for more efficient absorption and utilization in animals [1,2,3]. In addition, ZnO NPs not only exhibit strong antibacterial activity but also effectively regulate the immune system, promote growth, and enhance animals’ tolerance to oxidative stress [4,5]. These characteristics make ZnO NPs an ideal candidate for replacing antibiotics, particularly in reducing antibiotic use, improving animal health, and enhancing production performance [6,7].

While this review emphasizes biosynthesized ZnO NPs for animal nutrition, their synthesis method critically impacts applicability. Biosynthesis offers environmental advantages through green chemistry and may impart bioactive surface modifications. However, it typically yields NPs with lower production, reduced stability, and colloidal handling requirements, contrasting with conventional methods’ higher yields and particle control [8]. Despite these challenges, biosynthesized NPs’ unique properties drive optimization efforts. Key differences in properties and functionality will be detailed in Section 2.1 and Section 2.2.

Beyond their advantages in improving animal performance and health, ZnO NPs also show potential for environmental sustainability. Compared to traditional zinc supplements, ZnO NPs have higher bioavailability, which means that they can meet animals’ zinc requirements at lower doses, thereby reducing excessive zinc discharge into the environment and mitigating ecological risks [9].

However, despite the promising prospects of ZnO NPs in animal nutrition, there are still gaps in the current research. First, the optimization of ZnO NP dosage remains to be addressed, ensuring that their use improves animal performance while avoiding the negative effects of overuse. Secondly, there is a lack of sufficient studies on the long-term safety of ZnO NPs, particularly regarding the potential risks to animal health with prolonged exposure. Furthermore, the behavior, ecological toxicity, and mechanisms of ZnO NPs in the environment have yet to be fully explored, which are key factors determining their feasibility and sustainability in practical applications.

Therefore, this paper aims to explore the potential of ZnO NPs in animal nutrition, with a focus on their antibacterial, immune-regulatory, and growth-promoting effects. Additionally, it highlights the current research gaps and challenges, providing a reference for future studies in this area.

## 2. Green Synthesis and Physicochemical Tailoring of ZnO NPs

### 2.1. Green Synthesis Methods (Plant-/Microbe-Mediated) and Their Environmental Benefits

**Figure 1:**
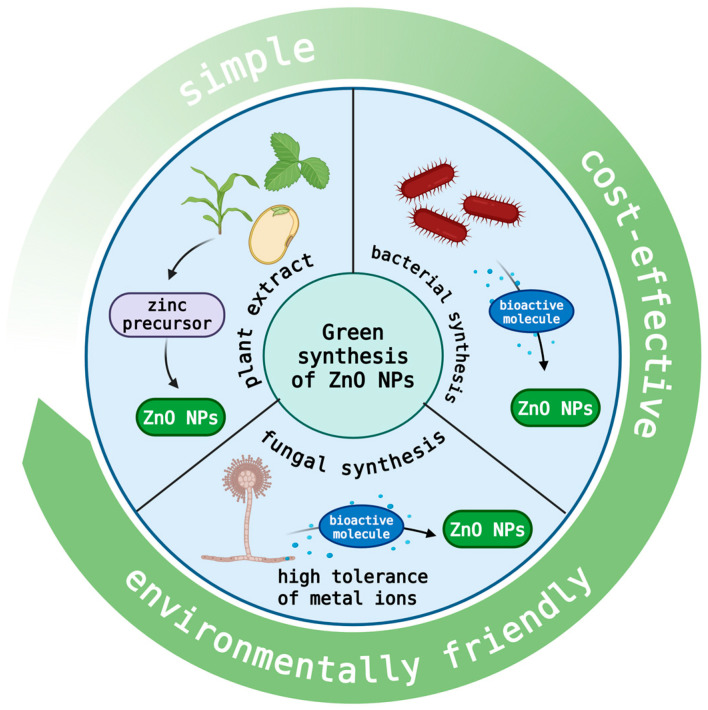
Green synthesis of nano-zinc oxide and its advantages.

Compared with traditional chemical synthesis methods, green synthesis not only reduces the use of harmful chemical reagents, but also significantly reduces the environmental impact, which has obvious advantages in terms of environmental protection and sustainable development. The green synthesis method for ZnO nanoparticles, using plant or microbial extracts (such as bacteria and fungi) as stabilizers to effectively reduce environmental toxicity, is shown in Figure 1. These natural extracts are rich in phenols, alcohols, and terpene compounds, which help to stabilize the nanoparticles and promote the synthesis of high-quality, stable ZnO nanoparticles [10,11]. Plant extracts, bacteria, and fungi can efficiently synthesize ZnO nanoparticles after reacting with zinc precursors and bioactive molecules. However, it is important to note that the chemical properties of ZnO NPs obtained via these green methods can vary significantly depending on the biological source used. For example, plant extracts and microorganisms (bacteria and fungi) provide diverse organic molecules that ‘decorate’ the surface of the nanoparticles, which in turn affects their chemical properties and, likely, their biological activities. This chemical diversity should be carefully considered, as it may influence the NPs’ antimicrobial efficacy, immune modulation, and other biological functions. Complete chemical characterization of the synthesized nanoparticles (NPs) is essential to understand their composition and behavior. Techniques like spectroscopy, chromatography, and surface charge analysis help identify the functional groups and chemical signatures on the nanoparticle surfaces. Standardizing the synthesis process is also important to ensure consistency between batches, ensuring reproducibility and reliable results in both research and practical applications such as animal feed additives or therapeutic agents.

### 2.2. Comparison of ZnO Nanoparticles with Conventional Zinc Oxide: Bioavailability, Ecotoxicity, and Environmental Impact

Zinc oxide nanoparticles (ZnO NPs) possess distinctive physicochemical properties, particularly their nanoscale dimensions (1–100 nm) and exceptionally high surface-to-volume ratio, which collectively contribute to their superior bioavailability when compared to conventional zinc oxide and organic zinc compounds [12]. The unique nanoscale characteristics of ZnO NPs facilitate enhanced permeability across biological barriers, enabling efficient penetration into cellular and tissue structures. This exceptional property renders them highly advantageous for diverse applications, including targeted drug delivery systems, antimicrobial formulations, and innovative agricultural solutions. In contrast, conventional zinc oxide, despite its effectiveness in specific applications, often necessitates higher concentrations to achieve comparable efficacy. Moreover, the synthesis processes of traditional zinc oxide may yield toxic by-products, posing significant risks of environmental contamination and adverse effects on non-target organisms [13]. The controlled release mechanism of Zn^2+^ ions from ZnO NPs represents a significant advancement in environmental safety, as it effectively regulates zinc ion availability, thereby minimizing the risk of excessive zinc accumulation in ecosystems and substantially reducing potential ecological toxicity [13]. Furthermore, recent developments in green synthesis methodologies utilizing plant extracts or microbial systems have demonstrated promising results in producing ZnO NPs with reduced ecotoxicity. These environmentally benign synthesis approaches not only mitigate pollution but also enhance the biodegradability of nanoparticles, resulting in significantly lower ecological impact compared to conventional zinc sources [10,11].

## 3. ZnO NPs as Multifunctional Feed Additives: Mechanisms and Applications

### 3.1. Antibacterial Action

**Figure 2:**
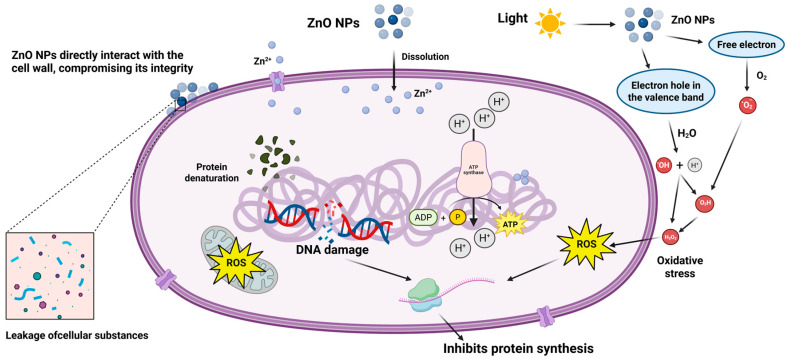
Schematic representation of the antibacterial mechanisms of ZnO nanoparticles against bacterial cells, illustrating key processes such as membrane disruption, Zn^2+^ ion uptake leading to cellular energy depletion and DNA damage, reactive oxygen species (ROS) generation, and photocatalytic ROS production under UV light.

Studies have shown that ZnO NPs inhibit the growth of *Escherichia coli* and *Klebsiella pneumoniae* at a concentration of 30 μg/mL, which is the highest antibacterial activity [14]. Although the antimicrobial activity of ZnO nanoparticles (NPs) is widely recognized, the exact mechanisms underlying their toxicity remain not fully elucidated and continue to be debated. ZnO NPs exhibit broad-spectrum antibacterial activity against both Gram-positive and Gram-negative bacteria, with a notably stronger inhibitory effect on Gram-negative strains, extending even to heat-resistant and pressure-tolerant spores [15,16]. Nanoparticles with antimicrobial properties can penetrate the peptidoglycan layer and cause significant damage to bacterial cells. The presence of the lipopolysaccharide layer in the bacterial cell wall enhances direct contact between the bacterial membrane and nanoparticles. This interaction occurs because lipopolysaccharides carry a negative charge, attracting and promoting the absorption of metal ions from positively charged nanoparticles, ultimately leading to bacterial cell damage [17]. Recent research indicates significant differences in sensitivity based on bacterial type: Gram-negative pathogens such as *E. coli* and *Pseudomonas aeruginosa* exhibit 100% growth inhibition (IC_100_) at 0.6 mM ZnO NPs, whereas Gram-positive strains, including *Staphylococcus* aureus and *Bacillus subtilis*, require higher concentrations (~1.0 mM) for equivalent inhibition [18]. Critically, exposure to these inhibitory concentrations for as little as 15 min induces substantial membrane damage in over 70% of bacterial cells, demonstrating rapid bactericidal action through membrane targeting. Several mechanisms have been proposed, including physical interactions between ZnO nanoparticles and the bacterial cell wall [19], the generation of reactive oxygen species (ROS) [20,21], and the release of free radicals and Zn^2+^ ions [22]. As illustrated in (Figure 2), the general mechanisms of ZnO nanoparticle antimicrobial action can be summarized as follows:

(1) Nanoparticles aggregate on the cell surface, causing membrane rupture and leakage of cellular contents [23,24].

(2) Bacterial cells absorb the released Zn^2+^ ions, leading to a significant decrease in cellular energy levels and DNA damage due to the loss of energy-rich molecules [25].

(3) ROS generation damages cellular components, thereby impairing essential cellular functions [26].

Among all inorganic photocatalytic materials, ZnO stands out due to its high photocatalytic efficiency, particularly its ability to strongly absorb ultraviolet (UV) light [27]. ZnO exhibits excellent responsiveness to UV light, leading to a significant enhancement in electrical conductivity, which notably facilitates its interaction with bacteria. In aqueous solutions, ZnO nanoparticles (NPs) exhibit phototoxicity under UV exposure, generating reactive oxygen species (ROS) such as hydrogen peroxide (H_2_O_2_) and superoxide anions (O2−) [28]. Ultimately, the bacterial cell membrane barrier is compromised by H_2_O_2_, leading to the disruption of cellular components such as DNA, proteins, and lipids, which results in cell damage and death. This process has driven the use of ZnO NPs in numerous antibacterial applications within the fields of biotechnologies and biomedical nanotechnology.

The antimicrobial efficacy of ZnO nanoparticles is influenced by various factors, including size, shape, zeta potential, surface morphology, surface defects, charge, functionalization, and other physicochemical properties. Additionally, environmental factors, microbial type, exposure time, and growth medium significantly affect the antibacterial activity of ZnO nanoparticles [29]. Some studies suggest that increased surface area-to-volume ratio, ligand deficiency, and enhanced surface energy contribute to the accumulation of metal oxide nanoparticles on the cell surface [30]. These studies have shown that the antibacterial properties of nanoparticles can be significantly improved by changing their surface properties, which provides new ideas for the development of more effective antibacterial agents. In addition, this discovery has also promoted the application of nanotechnology in agriculture, food safety, and medical fields, especially in terms of disease prevention as a feed additive, which is of great practical significance.

### 3.2. Anti-Inflammatory and Antioxidant Regulation

#### 3.2.1. Anti-Inflammatory Effects: Reduction of Inflammatory Reactions and Tissue Damage

**Figure 3:**
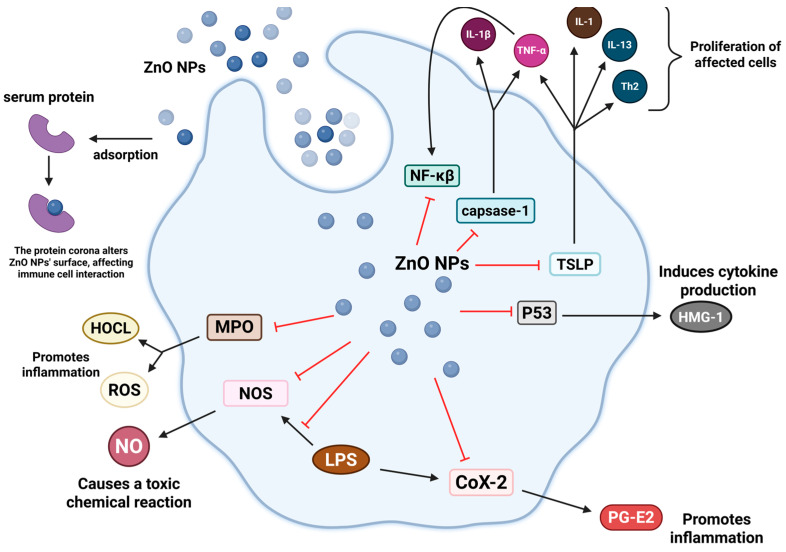
Key anti-inflammatory mechanisms mediated by ZnO nanoparticles, including NF-κB pathway modulation, mast cell regulation, and inflammatory enzyme inhibition. Black arrows indicate promotion or activation of cytokines/proteins, while red arrows indicate inhibition or suppression.

ZnO NPs have demonstrated significant anti-inflammatory potential through multiple molecular pathways, primarily by modulating key inflammatory mediators and signaling cascades. An overview of the different anti-inflammatory mechanisms adopted by ZnO NPs is shown (Figure 3). The nanoscale characteristics of ZnO NPs, particularly their high surface area-to-volume ratio, enhance their interaction with cellular components compared to bulk materials, leading to more effective suppression of inflammatory responses [31]. In addition, the interaction of zinc oxide nanoparticles (ZnO NPs) with serum proteins forms protein crowns, which regulate their interaction with cells and immune responses, thereby affecting their anti-inflammatory effects [32].

The anti-inflammatory activity of ZnO nanoparticles is regulated by three main mechanisms: (1) regulation of cytokine production and the NF-κB signaling pathway, (2) inhibition of the degranulation of mast cells, and (3) modulation of inflammatory enzyme activity. In the NF-κB pathway, ZnO NPs effectively inhibit LPS-induced activation by upregulating A20 expression and preventing nuclear translocation of NF-κB p65 in macrophages, while maintaining cytoplasmic levels of IκBα [33].

Regarding mast cell regulation, ZnO NPs demonstrate inhibitory effects by reducing thymic stromal lymphopoietin (TSLP) production and modulating p53 protein levels, thereby controlling the release of pro-inflammatory mediators such as IL-13 and HMGB1 [31]. Furthermore, ZnO NPs exhibit dose-dependent inhibition of key inflammatory enzymes, including COX-2 and iNOS. This enzymatic regulation prevents the production of inflammatory prostaglandins (PGE2) and excessive nitric oxide, which are associated with tissue damage and inflammation amplification [34,35,36].

ZnO NPs also regulate inflammation by reducing the production of NO. NO is a gas molecule produced by nitric oxide synthase (NOS) and plays an important role in many inflammatory diseases. ZnO NPs have been shown to effectively inhibit the expression of NOS and reduce the production of NO, thereby reducing the inflammatory response caused by NO [36]. In addition, ZnO NPs inhibit the catalysis of H_2_O_2_ to hypochlorous acid by inhibiting the activity of myeloperoxidase (MPO), thereby reducing the production of ROS and nitrating agents [32].

The multifaceted anti-inflammatory properties of ZnO NPs, encompassing cytokine regulation, cellular proliferation control, and enzymatic activity modulation, position them as promising candidates for therapeutic applications in inflammation-related disorders. Their ability to simultaneously target multiple inflammatory pathways offers potential advantages over conventional anti-inflammatory agents.

#### 3.2.2. Antioxidant Properties: Reducing Oxidative Stress and Cell Damage

Zinc oxide nanoparticles (ZnO NPs) demonstrate significant antioxidant capabilities through multiple mechanisms, both in extracellular environments and intracellular systems. While direct intracellular reactive oxygen species (ROS) scavenging remains unconfirmed, ZnO NPs exhibit antioxidant activity primarily through the modulation of antioxidant enzyme systems [37].

In vitro studies utilizing DPPH assays have established the free radical scavenging capacity of ZnO NPs, with electron density transfer from oxygen to nitrogen atoms resulting in decreased absorbance at 517 nm [38]. This antioxidant activity appears to be concentration-dependent and may be enhanced by surface-adsorbed phytochemicals [39]. Comprehensive evaluations using standardized assays (TAC, TRP, DPPH, and ABTS) have consistently demonstrated concentration-dependent antioxidant activity, with particularly strong performance in ABTS assays [40,41,42,43,44,45].

In vivo investigations have further substantiated these findings. Dietary supplementation with ZnO NPs (39.2–49.11 nm) at concentrations ranging from 5 to 80 ppm has shown significant enhancement of antioxidant enzyme activities (SOD, CAT, and GPx) and a reduction in oxidative stress markers (MDA and lipid peroxides) in various animal models [46,47,48]. Notably, these studies have observed increased mRNA expression of key antioxidant enzymes (SOD1, CAT, GPX1, and GPX7) in multiple tissue types.

The antioxidant properties of ZnO NPs, combined with their antimicrobial and anti-inflammatory activities, contribute to their therapeutic potential in wound healing, immune modulation, and cellular protection. Table 1 provides a comprehensive overview of the mechanisms by which ZnO NPs exert these effects, highlighting their multifunctional roles in pathogen elimination, inflammation reduction, and oxidative stress mitigation.

### 3.3. Growth Performance and Product Quality Improvements

In the domain of animal production, zinc oxide nanoparticles (ZnO NPs) have emerged as a significant additive, owing to their distinctive physicochemical characteristics. These particles have been the focus of extensive research and application due to their antimicrobial, antioxidant, immunomodulatory, and growth-promoting properties. A substantial body of research has demonstrated that the judicious administration of ZnO NPs has the capacity to enhance immune function in animals, thereby promoting enhanced productivity and the quality of meat, eggs, milk, and other products. This is achieved by means of improving intestinal health and promoting nutrient absorption.

The specific application effects and research progress of ZnO NPs in different animal species are listed in Table 2, covering a wide range of animal models, such as cattle, sheep, pigs, and poultry. From the experimental results, the application of ZnO NPs showed significant positive effects in different animals, especially in disease resistance, immune enhancement, and growth promotion. Furthermore, the bioavailability and environmental impact of ZnO NPs have garnered mounting interest, with future research potentially focusing on optimizing their dosage, enhancing their environmental sustainability, and reducing potential toxic effects.

#### 3.3.1. Therapeutic Effect

Zinc oxide nanoparticles (ZnO NPs) exhibit superior therapeutic efficacy compared to conventional zinc sources, which is attributed to their nanoscale dimensions, high surface area, and enhanced bioavailability [58,59]. These properties enable effective antimicrobial action at lower concentrations while maintaining environmental compatibility.

The antimicrobial mechanism of ZnO NPs involves membrane disruption, leading to cellular content leakage and bacterial death [60]. Their efficacy against both Gram-positive and Gram-negative bacteria is well-documented, with size-dependent activity observed against Staphylococcus aureus and concentration-dependent inhibition of *Escherichia coli* O157 [16,61].

In veterinary applications, ZnO NPs demonstrate broad-spectrum activity against gastrointestinal pathogens. Notably, they exhibit dose-dependent antifungal effects against Candida albicans, including fluconazole-resistant strains, at concentrations of 119 μg/mL [62,63,64]. For reproductive health, biosynthesized ZnO NPs have shown remarkable efficacy in treating postpartum endometritis in dairy cows, significantly reducing uterine pathogens and improving reproductive outcomes [52].

In poultry production, ZnO NPs effectively combat respiratory pathogens when combined with tylosin, while reducing antibiotic residues [65]. Their antiparasitic properties have been demonstrated against Eimeria flexneri in broilers and Toxoplasma gondii in murine models, showing reduced pathogen load and improved tissue integrity [66,67].

These findings position ZnO NPs as promising therapeutic agents for various veterinary applications, offering advantages in efficacy, reduced dosage requirements, and minimized resistance development.

#### 3.3.2. Immunomodulatory Effects of Zinc Oxide Nanoparticles in Animal Production

##### Terrestrial Livestock Applications

Zinc oxide nanoparticles (ZnO NPs) serve as potent immunomodulators in terrestrial livestock, enhancing both innate and adaptive immunity through multiple mechanisms. Their nanoscale properties (high surface area and bioavailability) improve zinc utilization efficiency compared to conventional sources, addressing zinc deficiency-related immune dysfunction [68,69]. In swine production, dietary supplementation with 800 mg/kg ZnO NPs increases average daily weight gain by 18%, reduces diarrhea incidence by 40%, and enhances antioxidant capacity through elevated serum SOD (↑32%) and GSH-Px (↑25%) activities [49,70]. The nanoparticles strengthen intestinal barrier integrity via upregulation of tight junction proteins (ZO-1 and occludin) and modulate gut microbiota composition, suppressing pathogenic *Escherichia coli* K88 while promoting beneficial microbial communities [71,72].

Poultry studies demonstrate ZnO NPs’ dual role in immune organ development and pathogen control. Supplementation increases thymus (↑15%), bursa (↑12%), and spleen (↑9%) weights, correlating with enhanced antimicrobial activity against Salmonella enterica and *Escherichia coli* O157 [73,74,75,76]. The size-dependent antibacterial efficacy (40–60 nm particles showing optimal performance) arises from membrane disruption and reactive oxygen species generation [19,77]. Furthermore, ZnO NPs regulate stress-responsive genes, modulating HSP70/90 expression in intestinal tissues and appetite-related hormones (CCK and gastrin), thereby improving stress resilience [78].

##### Aquatic Species Applications

In aquaculture systems, ZnO NPs exhibit superior immunostimulatory effects over bulk zinc, achieving equivalent biological responses at 60% lower dosages (40–60 mg/kg feed) [79,80,81]. Evidence suggests that ZnO NPs enhance the immune response in aquatic animals by modulating cytokine gene expression and promoting non-specific receptor-mediated cell development, including neutrophils. This creates a strong first line of defense against infections and foreign pathogens, such as bacteria [79,82,83]. Furthermore, ZnO NPs have demonstrated potent antimicrobial activity at low doses. Studies have shown that the addition of 40–60 mg/kg of ZnO NPs to fish feed significantly improves immune performance and disease resistance, effectively preventing infections [51,84,85]. Their eco-friendly profile minimizes zinc accumulation in aquatic ecosystems while maintaining therapeutic efficacy. This immunomodulatory capacity, coupled with growth-promoting and antimicrobial properties, positions ZnO NPs as multifunctional additives for sustainable animal production systems.

#### 3.3.3. Growth Promotion and Productivity Improvement

In recent years, the incorporation of nanotechnology, particularly zinc oxide nanoparticles (ZnO NPs), into animal feed has attracted considerable attention. These nanoparticles have been shown to enhance the bioavailability of trace minerals, with notable benefits observed in weaned piglets and poultry. ZnO NPs not only support improved growth and development but also enhance feed conversion efficiency, leading to significant economic advantages [86,87].

##### Growth Promotion Mechanisms and Feed Efficiency Optimization

A study by Qu demonstrated that supplementing piglet diets with small-sized zinc oxide nanoparticles (ZnO NPs, average size: 21 nm) significantly improved daily weight gain, the feed conversion ratio (FCR), and the lymphoid organ index. Additionally, ZnO NPs enhanced intestinal health, as evidenced by increased villus height and a higher villus height-to-crypt depth (VH/CD) ratio [77]. Furthermore, ZnO NPs have been shown to reduce somatic cell counts and increase milk production in cows suffering from subclinical mastitis, indicating their potential as both a preventive and therapeutic agent for managing this condition in dairy cows [88].

In vitro studies have also highlighted the effects of ZnO NPs on rumen microorganisms. The addition of ZnO NPs (100–200 mg/kg) to rumen contents during the early stages of incubation (6 to 12 h) promoted microbial growth, enhanced microbial protein synthesis, and improved energy utilization efficiency. Specifically, these nanoparticles increased concentrations of volatile fatty acids, microbial crude protein production, and organic matter fermentation. However, they also led to higher ammonia–nitrogen concentrations and altered the acetic acid-to-propionic acid ratio [89].

##### Improving Poultry Performance

The use of zinc oxide nanoparticles (ZnO NPs) in poultry production has shown considerable benefits. Broiler diets often contain high levels of phytates, which can impair zinc absorption [90]. In comparison with both organic and inorganic zinc sources, the addition of 80 mg/kg of ZnO NPs has proven to be the most effective in improving broiler performance and immune function [91]. A study by Abeer demonstrated that supplementing broiler diets with 90 mg/kg of ZnO NPs significantly increased body weight and sustained weight gain throughout the trial, outperforming conventional bulk zinc oxide [92].

Furthermore, the inclusion of 20–60 mg/kg of ZnO NPs in laying hen diets enhanced nutrient digestibility and improved liver and kidney function [93]. In broiler chickens, supplementation with 30–90 mg/kg of ZnO NPs led to notable increases in weight gain, feed intake, and overall growth performance [73]. Ahmed’s research found that adding 20, 40, and 60 mg/kg of ZnO NPs to broiler rations under hot environmental conditions improved growth performance, nutrient digestibility, carcass quality, and liver and kidney function [94]. Similarly, Abbasi reported that ZnO NP supplementation significantly boosted quail fertility, with peak egg production achieved at dietary zinc levels of 67–72 mg/kg, whether using ZnO NPs or zinc oxide microparticles [53].

##### Effects on Meat and Egg Quality

Zinc oxide nanoparticles (ZnO NPs) demonstrate enhanced bioavailability and reduced dosage requirements compared to conventional zinc supplements. Studies demonstrate that ZnO NPs exert beneficial effects on multiple poultry performance parameters, including carbonic anhydrase activity optimization, feed conversion efficiency enhancement, and improvements in egg quality indices [95,96,97]. Significant increases in serum zinc levels, eggshell zinc deposition, and tibial bone mineralization have been documented [98], along with enhanced antioxidant capacity in biological systems [96,99]. Dietary supplementation with ZnO NPs has been demonstrated to enhance eggshell strength, intensify yolk coloration, and elevate egg zinc content, thereby improving nutritional value [100].

Safdar conducted a comparative analysis of ZnO NPs synthesized through various green chemistry approaches, evaluating their effects on laying hen productivity, skeletal integrity, and oxidative stress responses. While nanoparticle synthesis methods showed no significant performance differences, optimal production parameters were achieved at a 70 mg/kg ZnO NP concentration [101]. Complementary research by Usama revealed that broiler diets supplemented with 20 mg/kg ZnO NPs significantly improved carcass characteristics, including enhanced dressing percentage, edible meat yield, and total carcass weight [102,103,104,105].

In porcine models, ZnO NP supplementation improved meat quality through enhanced water retention capacity. These improvements were mechanistically linked to Nrf2-GCL pathway activation, which mitigates oxidative damage while improving myofibrillar structure and tissue integrity [106].

Despite these advantages, emerging evidence indicates that high concentrations of nanometer-scale zinc oxide may raise toxicological concerns. Cytotoxicity assessments in avian models have demonstrated a dose-dependent relationship between exposure and hematological abnormalities, including erythrocyte nuclear mutations and binucleated cell formation [107]. Contrastingly, Yusuf reported decreased bone mineralization indices and compromised eggshell quality in nano-ZnO groups, suggesting potential interference with calcium–phosphate metabolism [108]. These findings underscore the critical balance between ZnO NPs’ zootechnical benefits and their toxicological profile, emphasizing the necessity for precise dosage optimization to prevent counterproductive effects on poultry growth performance and product quality.

### 3.4. Reproductive Health and Genetic Enhancement

A major challenge in livestock production is the limited availability of high-quality breeding stock, where the presence of genetically superior reproductive males can significantly enhance overall herd productivity. Zinc plays a crucial role in sperm motility by regulating the ATP system within phospholipid energy reserves, while its deficiency increases oxidative stress, leading to diminished sperm quality. Moreover, oxidative damage caused by reactive oxygen species during sperm cryopreservation can impair motility, an effect that can be effectively mitigated using antioxidant nanoparticles such as zinc oxide nanoparticles (ZnO NPs). Abedin demonstrated that ZnO NPs more effectively prevented oxidative damage to stag spermatozoa compared to selenium nanoparticles (Se-NPs) and improved semen quality by upregulating HSP gene expression [109]. Additionally, ZnO NPs synthesized by Leila from mulberry–orange fruit extracts significantly enhanced the survival, viability, and DNA integrity of ram spermatozoa during cryopreservation at 4 °C [110]. Motlagh further reported that supplementing sperm storage media with 100 μg/mL of zinc nanoparticles and ZnO NPs successfully preserved the quality of rooster semen during the cooling period [111].

## 4. Dissolution and Bioavailability of Zinc Oxide Nanoparticles in Acidic Environments

When adding zinc oxide nanoparticles (ZnO NPs) to animal feed, controlling dissolution kinetics presents a challenging issue. ZnO NPs exhibit unique dissolution properties in acidic environments, such as the gastrointestinal (GI) tract, where the pH typically ranges from 1 to 4, significantly affecting their bioavailability. This dissolution is influenced by various factors, including the particle size, specific surface area, and aggregation state of the zinc oxide nanoparticles [112]. Smaller particle sizes and larger surface areas typically increase the dissolution rate, as they provide more surface area for interaction with the acidic medium. However, altering the feed matrix can affect the dissolution of ZnO in the gastrointestinal tract. The presence of various biological matrices such as proteins, carbohydrates, and lipids may influence the dissolution process. Depending on their chemical nature and interactions with the nanoparticles, these matrices can either promote or hinder the release of zinc ions. For example, circular dichroism studies found that the conformation of ZnO NPs changed after interaction with skim milk and casein. After 1 min of exposure to ZnO NPs, slight deformation of the helical bands was observed, and after 168 h of exposure, no significant deformation was observed, indicating that proteins can bind to zinc oxide nanoparticles and alter their surface properties, thus influencing the dissolution rate and subsequent bioavailability [113]. Furthermore, bioavailability is not solely dependent on the dissolution rate. Other factors, including the presence of competing minerals such as calcium or phytates, can also affect the efficiency of zinc absorption [114]. Therefore, understanding how these factors interact to influence the dissolution and absorption processes of zinc oxide nanoparticles is crucial for optimizing their use in animal feed.

## 5. Toxicity and Safety Considerations

Although the addition of metal nanoparticles to animal diets has potential applications, the long-term effects of their use as feed additives remain largely unknown. At present, the toxicological data on nanoparticles are incomplete and their potential hazards need to be thoroughly assessed. Therefore, before nanoparticles can be officially recommended for use in animal nutrition, extensive long-term animal studies must be conducted to evaluate their toxicity characteristics and establish a safe range of use to ensure their safety and reliability in the livestock industry. Recent advances in omics approaches, such as transcriptomics, proteomics, and metabolomics, offer powerful tools to unravel the molecular mechanisms of nanoparticle toxicity. These methods provide comprehensive insights into gene expression changes, protein dysregulation, and metabolic pathway alterations, enabling a deeper understanding of nanomaterial interactions with biological systems beyond traditional cytotoxicity assays [115]. For instance, transcriptomic studies (e.g., RNA-Seq) in hepatic cells have shown that ZnO NPs significantly upregulate and downregulate genes involved in immune responses and oxidative stress, while metabolomic analyses in zebrafish models reveal disruptions in the tricarboxylic acid cycle and oxidative phosphorylation, leading to ATP loss and energetic stress. These omics techniques have identified key biomarkers, such as reactive oxygen species (ROS) and inflammatory cytokines, which mediate ZnO NP-induced damage and highlight dose-dependent effects [115].

Abdelnour’s study found that 40 mg/kg ZnO NPs significantly increased creatinine levels and the activity of AST, ALT, and CK-MB, suggesting that 40 mg/kg ZnO NPs may have toxic effects on multiple organ systems, including the kidneys, liver, and heart, leading to organ dysfunction [116]. Other research has shown that ZnO NPs, through the release of Zn^2+^ ions, significantly reduced the survival rate of A172 cells within 24 h, induced DNA damage, and significantly inhibited the movement of zebrafish embryos, suggesting that Zn^2+^ ions play a key role in their cytotoxicity, genotoxicity, and behavioral toxicity [117]. These findings highlight the need for caution in the use of nanoparticles in animal feed and emphasize the need for further safety evaluations. The integration of omics data, particularly proteomics and lipidomics, has further elucidated that ZnO NPs disrupt protein homeostasis and lipid metabolism in macrophages, contributing to inflammatory responses and cellular damage [115]. Such mechanistic insights are crucial for developing safer nanoparticle formulations and refining dosage regimens to mitigate environmental and health risks.

**Figure 4:**
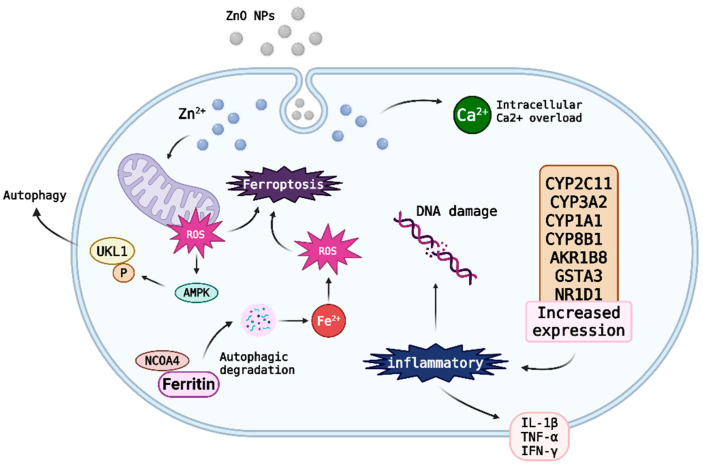
Key toxicity mechanisms of ZnO nanoparticles, including cellular dissolution, ROS generation, inflammatory activation, genetic alterations, and ferroptosis.

The mechanism of toxicity of ZnO nanoparticles is shown in Figure 4 and mainly involves oxidative stress and the production of reactive oxygen species leading to cell damage [118]. ZnO NPs dissolve in acidic environments, releasing Zn^2+^ ions which, together with their semiconducting properties, increase intracellular oxidative stress [118]. This in turn causes DNA and mitochondrial damage, ultimately leading to cell apoptosis or necrosis [118]. In addition, ZnO NPs can activate inflammatory responses, increasing the expression of inflammatory factors such as IL-1β, TNF-α, and IFN-γ, which exacerbate tissue damage [118]. They also alter gene expression related to metabolism, repair, and immune responses, including upregulation of the CYP450 family of enzymes, further enhancing cytotoxic effects [118]. ZnO NPs may also induce a novel mechanism of vascular endothelial cell damage through ferritin autophagy and ferroptosis, in which the AMPK-ULK1 pathway plays a critical role [119]. In particular, NCOA4-mediated ferritin autophagy degradation is a key step in ZnO NP-induced ferroptosis [119].

## 6. Environmental Fate and Ecotoxicity of Zinc Oxide Nanoparticles in Animal Feed

As the use of ZnO NPs in animal feed continues to increase, it is crucial to assess their long-term ecological risks. Once incorporated into animal diets, ZnO NPs are typically excreted, with a significant portion entering the environment, posing potential threats to aquatic and soil ecosystems. Upon release into the environment, ZnO NPs undergo various transformations, such as dissolution, aggregation, or sedimentation. These processes are influenced by factors such as pH, ionic strength, and dissolved organic matter [120]. Smaller ZnO NPs typically exhibit higher bioavailability and greater toxicity to aquatic organisms. Moreover, aggregation and coagulation in aquatic environments can alter the size distribution of ZnO NPs, subsequently affecting their toxicity [121].

The ecotoxicity of ZnO NPs in aquatic ecosystems has been well-documented. Studies show that ZnO nanoparticles can induce oxidative stress, leading to tissue damage, immune responses, and developmental abnormalities in aquatic organisms [121]. Additionally, the bioaccumulation of ZnO NPs within organisms is influenced by nanoparticle concentration, exposure duration, and particle size [122]. Smaller nanoparticles are more readily absorbed by organisms, resulting in higher bioaccumulation and toxicity. These factors must be considered when evaluating the potential environmental impacts of ZnO nanoparticles.

Although ZnO NPs may offer benefits in terms of livestock growth and antimicrobial activity, careful management of their environmental impact is essential to prevent the accumulation of toxic zinc in soil and water. Future research should focus on the environmental behavior of ZnO NPs, particularly their transformation processes and bioaccumulation, in order to develop sustainable strategies for their use in animal feed.

## 7. Regulatory Landscape and Mitigation Strategies

Current regulatory frameworks for zinc supplementation in animal feed lack specific provisions addressing the unique properties of zinc oxide nanoparticles (ZnO NPs), despite evidence of their distinct ecotoxicological profiles compared to conventional zinc compounds. Specific regulatory standards are detailed in Table 3. To bridge this regulatory gap and mitigate risks associated with ZnO NPs, a multi-faceted strategy is essential. Dosage optimization must be prioritized, as studies indicate that high concentrations (>40 mg/kg) induce organ toxicity—evidenced by elevated serum creatinine, AST, ALT, and CK-MB levels in livestock—while lower doses sustain growth promotion and antimicrobial efficacy without adverse effects. Combining ZnO NPs with conventional zinc sources further reduces nephrotoxicity and hepatotoxicity risks. Long-term safety assessments should integrate advanced omics approaches (transcriptomics, proteomics, and metabolomics) to identify toxicity biomarkers, such as dysregulation of the AMPK-ULK1 pathway and NCOA4-mediated ferritin autophagy, which drive ferroptosis and vascular endothelial damage. Concurrently, environmental impact mitigation requires closed-loop waste management systems to recover excreted nanoparticles via membrane filtration, preventing aquatic ecosystem disruption, as ZnO NPs exhibit size-dependent bioaccumulation and induce oxidative stress in organisms like zebrafish. Standardization of green synthesis protocols is critical to minimize batch variability; physicochemical characterization (size: ±5 nm, zeta potential: ±10 mV) and surface functionalization using plant-derived stabilizers (e.g., mulberry extracts) enhance nanoparticle stability while reducing ecotoxicity [10,110]. Regulatory bodies must enforce traceability systems (e.g., isotopic labeling with ^68^Zn) and real-time monitoring technologies to align ZnO NP applications with One Health principles, ensuring sustainable adoption in global animal production systems [114].

## 8. Summary and Future Prospects

Zinc oxide nanoparticles (ZnO NPs) have emerged as a promising alternative to traditional feed additives in animal nutrition, offering significant antimicrobial, growth-promoting, and immune-regulatory benefits. Their unique physicochemical properties, such as nanoscale size and high surface area, contribute to their effectiveness in improving animal health and productivity. ZnO NPs have shown strong antibacterial activity, promoting growth and enhancing the immune response in livestock, thus providing a viable solution to the growing concerns over antibiotic resistance in animal production.

Furthermore, ZnO NPs have demonstrated their potential in improving meat, egg, and milk quality, as well as promoting better nutrient absorption and intestinal health. The green synthesis methods used for ZnO NP production have also garnered attention, as they provide an environmentally friendly approach with reduced toxicity, thereby contributing to more sustainable agricultural practices.

However, despite the promising applications of ZnO NPs in animal feed, several key challenges remain. First, the optimization of their dosage is critical to ensure that the benefits of ZnO NPs are maximized without causing adverse effects due to overuse. Additionally, long-term safety studies are still lacking, particularly regarding potential risks to animal health with prolonged exposure to ZnO NPs. Furthermore, the ecological toxicity and environmental behavior of ZnO NPs have not been fully explored, and more research is needed to understand their fate and impact on the environment.

Looking ahead, ZnO NPs hold great promise for revolutionizing animal nutrition and supporting sustainable livestock production. However, future research should focus on optimizing their dosage, enhancing bioavailability, and investigating the mechanisms underlying their antimicrobial efficacy. Moreover, understanding the environmental implications and ensuring the safe, sustainable use of ZnO NPs in animal feed will be essential for their continued application in the livestock industry. Developing safe and effective regulatory frameworks will be crucial to ensure their responsible use and mitigate any potential ecological risks.

## Figures and Tables

**Table 1:** Mechanisms of antibacterial, anti-inflammatory, and antioxidant actions of ZnO NPs.

Function	Mechanism	Description
Antibacterial	ROS Generation	ZnO NPs produce reactive oxygen species, damaging bacterial cell membranes and inhibiting bacterial growth.
	Zn^2+^ Ion Release	Zn^2+^ ions penetrate bacterial cells, disrupting metabolic functions and inhibiting protein and nucleic acid synthesis, ultimately causing cell death.
	Particle Size Effect	The nanoscale size of ZnO NPs increases surface area, enhancing interaction with bacteria and boosting antibacterial activity.
	Bacterial Membrane Disruption	ZnO NPs disrupt bacterial membrane integrity, leading to the leakage of cell contents and cell death.
Anti-inflammatory	Inhibition of Pro-Inflammatory Factors	ZnO NPs inhibit the generation of pro-inflammatory factors (e.g., TNF-α and IL-6), reducing the inflammatory response.
	Reduction of Tissue Damage	By reducing free radical production, ZnO NPs mitigate tissue damage caused by inflammation, protecting cells.
	Blocking of Cytokine Signaling	ZnO NPs block pathways such as NF-κB, reducing the production of inflammatory cytokines and alleviating inflammation.
	Reduction in Inflammatory Enzyme Activity	ZnO NPs decrease the expression of enzymes such as COX-2 and iNOS, which are associated with the amplification of inflammation.
Antioxidant	Free Radical Scavenging	ZnO NPs eliminate free radicals, reducing damage to cellular membranes and structures caused by oxidative stress.
	Enhancement of Antioxidant Enzyme Activity	ZnO NPs activate antioxidant enzymes (e.g., SOD, CAT, and GSH-Px), neutralizing free radicals and increasing cellular antioxidant capacity.
	Reduction in Malondialdehyde (MDA) Levels	ZnO NPs reduce oxidative stress, leading to lower MDA levels, mitigating lipid peroxidation, and cellular damage.
	Strengthening Cellular Antioxidant Defense	ZnO NPs enhance intracellular antioxidant defense mechanisms, protecting cells from protein, DNA, and lipid damage.

**Table 2:** Biological effects of zinc oxide nanoparticles in animal models.

Animal Species	Sample Size	Trial Duration	Route of Administration	ZnO Nanoparticle Dosage	Observed Effects	References
Pigs	*n* = 4	14 days	Dietary supplementation	800 mg/kg	Improved growth performance, reduced diarrhea incidence, enhanced intestinal barrier function, modulated immune responses, decreased zinc excretion, promoted intestinal morphology	[49]
Broilers	*n* = 6	42 days	Dietary supplementation	40/60/100 mg/kg	Modulated appetite-related genes (ghrelin and CCK), regulated heat stress protein genes (HSP70 and HSP90)	[50]
Oreochromis niloticus	*n* = 15	8 weeks	Dietary supplementation	20/40/60 mg/kg	Enhanced antioxidant capacity, improved blood health, boosted immune function, optimized metabolic profiles, reduced liver damage, enhanced resistance to Staphylococcus aureus at 40 mg/kg dietary supplementation	[51]
Dairy Cattle	Total = 500	5 days treatment + 45–60 days observation	Intrauterine infusion	20 μg/mL (150 mL daily, total 3 mg/day per cow)	Effective clinical cure of multidrug-resistant uterine infections, induction of estrus and pregnancy, broad-spectrum antibacterial activity	[52]
Quails	*n* = 4	28 days	Dietary supplementation	0–130 mg/kg	Improved eggshell thickness, enhanced fertility, increased egg weight and eggshell surface, optimized egg production with reduced zinc requirement	[53]
Mice	*n* = 6	28 days	Intraperitoneal injection	5.6 mg/kg	Significantly increased dopamine levels in the brains of Parkinson’s disease mice, activated the expression of the dopa decarboxylase gene while maintaining tyrosine hydroxylase activity. It had no significant effect on norepinephrine or epinephrine levels, effectively improving motor dysfunction and reversing weight loss symptoms induced by rotenone	[54]
Rats	*n* = 7	42 days	Intraperitoneal injection	4 mg/kg	Attenuated oxidative damage, suppressed neuroinflammatory response, inhibited neuronal apoptosis, restored neurotransmitter balance, mitigated histopathological alterations	[55]
Lambs	STE: *n* = 9/group LTE: *n* = 7/group	42 days	Dietary supplementation	STE: 80 mg Zn/kg diet (ZnO-NPs, ZnP-NPs) LTE: 40 mg/kg (ZnO-NP40), 80 mg/kg (ZnO-NP80, ZnO-80)	In vitro: enhanced dry matter digestibility; short-term: reduced total bacterial population with ZnP-NPs; long-term: decreased ammonia-N concentration (ZnO-NP80), increased carboxymethyl cellulase and xylanase activities; histopathology: inflammatory changes in ruminal papillae and epithelium regardless of Zn form/dose/duration, improved feed-use efficiency via altered fermentation patterns enhancing SCFA transport capacity	[56]
Rabbits	*n* = 15	45 days	Dietary supplementation	Gp.3: 60 mg/kg ZnO NPs Gp.4: 30 mg/kg ZnO + 30 mg/kg ZnO NPs	Gp.3 (ZnO NPs): elevated liver enzymes (ALT/AST), hypoproteinemia/hypoalbuminemia, reduced lipid profile (TG/TC/HDL-C), increased renal markers (creatinine/urea), hepatic/renal oxidative stress (↑ MDA, ↓ CAT), leukocytosis/lymphocytosis, hepatic/renal DNA damage, severe hepatic hydropic degeneration and renal interstitial nephritis; Gp.4 (combined): mitigated adverse effects (less severe biochemical/oxidative/genotoxic changes and histopathology) while retaining zinc benefits	[57]

**Table 3:** Regulatory standards for ZnO NPs vs. zinc compounds.

Regulatory Body	Standard	NP-Specific Clause
EFSA (EU)	≤150 mg/kg total Zn in feed	Yes: Requires nano-specific bioavailability assessment (since 2021)
FDA (US)	GRAS status (21 CFR §573.920)	No: Applies to ZnO compounds generically
GB Standard (China)	120–250 mg/kg total Zn	Partial: Recommends NP accumulation monitoring
OECD	TG 305: Bioaccumulation test	Under revision: 2023 draft adds agreement on nanomaterials

## Data Availability

Data generated in this study are presented in the article.
